# Momilactones A, B, and Tricin in Rice Grain and By-Products are Potential Skin Aging Inhibitors

**DOI:** 10.3390/foods8120602

**Published:** 2019-11-21

**Authors:** Nguyen Van Quan, Dam Duy Thien, Tran Dang Khanh, Hoang-Dung Tran, Tran Dang Xuan

**Affiliations:** 1 Graduate School for International Development and Cooperation, Hiroshima University, Hiroshima 739-8529, Japan; nguyenquan26@gmail.com; 2 Dai Nam Manufacturing & Trade Co. Ltd., 7th District, Ngo Duc Ke Street No 57, Vung Tau City 78212, Vietnam; khaixuan.study@gmail.com; 3 Agricultural Genetics Institute, Pham Van Dong Street, Hanoi 122000, Vietnam; tdkhanh@vaas.vn; 4 Faculty of Biotechnology, Nguyen Tat Thanh University, Ho Chi Minh 72820, Vietnam; thdung@ntt.edu.vn

**Keywords:** momilactones A and B, tricin, antioxidants, anti-elastase, anti-tyrosinase, rice grains, rice by-products

## Abstract

We previously reported the inhibitory potentials of momilactones A (MA) and B (MB) against key enzymes related to type 2 diabetes and obesity. In this study, antioxidant and anti-skin-aging activities of MA and MB were investigated and compared with tricin, a well-known antioxidant and antiaging flavonoid in rice. MA, MB, and tricin were purified from rice husk by column chromatography and their biological activities were subsequently assayed by in vitro trials. The contents of MA, MB, and tricin of different commercial rice cultivars in Japan were quantified and confirmed by ultra-performance liquid chromatography-electrospray ionization-mass spectrometry (UPLC-ESI-MS) and high-performance liquid chromatography (HPLC) analyses. The antioxidant assays revealed a synergistic activity of the mixture MA and MB (MAB, 1:1, *v/v*). In addition, in 2,2’-azino-bis (ABTS) assay, IC_50_ values of MAB (0.3 mg/mL) and tricin (0.3 mg/mL) was 4-fold and 9-fold greater than that of individual MB (1.3 mg/mL) or MA (2.8 mg/mL), respectively. The in vitro enzymatic assays on pancreatic elastase and tyrosinase indicated that MA and MB were potential to relief skin wrinkles and freckles. In detail, MA exerted higher inhibition on both enzymatic activities (30.9 and 37.6% for elastase and tyrosinase inhibition, respectively) than MB (18.5 and 12.6%) and MAB (32.0 and 19.7%) at a concentration of 2.0 mg/mL. Notably, MA and the mixture MAB exhibited stronger inhibitions on elastase and tyrosinase in comparison with tricin and vanillin. MA, MB, and tricin in rice are potential to develop cosmetics as well as supplements for skin aging treatments.

## 1. Introduction

In humans, the skin is the most important organ which plays a crucial role as the first protective barrier. The skin protects our body from not only physical factors—such as collisions, clashes, UV radiation, etc.—but also from chemical agents, for instance, toxics, oxidants, or oxidative stresses. These factors may impact on many aspects of the skin function, especially on skin cells which are able to be dropped in an undesired condition of apoptosis [1]. Among skin injuries caused by UV radiation, the elasticity reduction or wrinkles and melanogenesis increases or freckles are the most detectable because they directly expose via visual phenotypes [2,3,4]. The stability of skin is maintained by elastin and collagen fibers while melanin is an important pigment that helps protect skin from UV radiation. Basically, wrinkles and freckles are associated with the surge of dermal enzymatic activities containing elastase and tyrosinase. While elastase acts as the elastin’s breaker, tyrosinase enhances the occurrence of melanin and consequently results in the formation of freckles. Besides, practical investigations reported that skin disorders and complications would sooner or later be developed in all diabetic and obesity patients [5,6,7]. These physiological disturbances are mainly attributed to the excess accumulation of reactive oxygen species (ROS) or oxidative stress which can influence biological macromolecule components and cellular functions, thus leading to aging-related disorders or melanogenesis [4]. Therefore, the identification of antioxidants and elastase and tyrosinase inhibitors is the most effective approach in the prevention of skin aging, wrinkling, and browning.

Rice (*Oryza sativa*) is a global crop which has a long history of safe usage as an indispensable food for human. The use of rice products in cosmeceuticals is not a novelty, of which, a diverse number of bioactive compounds has been identified as ferulic, γ-oryzanol, and members of flavonoids, flavones, etc. [8,9]. In fact, there are many cosmetic firms in the world producing those natural substances and benefiting greatly from selling such products originated from rice. Among rice’s flavonoids, tricin is currently attracting great interest thanks to its pharmaceutical benefits and physiological activities [10]. Tricin was reported to interfere with various serious human diseases, including skin-aging and cancers [11].

In previous studies, we discovered momilactones A (MA) and B (MB) as potential diabetic and obesity inhibitors in rice grain as well as in rice bran [12,13]. Their biological activities were also reviewed from literature containing allelopathic, antifungal, antibacterial, and anticancer activities [12]. However, the anti-skin-aging activity of MA and MB has not been studied so far. On the other hand, in previous work, we found that the use of SEP-PAK C18 cartridge might increase the sensitivity of MA and MB detections by high-performance liquid chromatography (UV–VIS detector) [14]. The application of more advanced techniques such as ultra-performance liquid chromatography (UPLC) integrated electrospray ionization-mass spectrometry (ESI-MS) has not been used in quantification of MA and MB in rice grains. In common with MA and MB, the tricin content in rice grains has been limitedly reported.

Hence, this research was conducted in order to investigate the antioxidant and anti-wrinkle and freckle properties of MA, MB, and tricin isolated from rice husks; and to validate the method of quantifying such bioactive metabolites in common rice grains. The outcomes may provide a good option in selecting the best rice grains which involve high amounts of beneficial molecules like momilactones and tricin that can simultaneously combat several chronic diseases as diabetes type 2, obesity, and skin problems.

## 2. Materials and Methods

### 2.1. Reagents and Pure Compounds

The extraction and isolation solvents were purchased from Junsei Chemical Co., Ltd., Tokyo, Japan and Fisher Scientific company, Hampton, NH, USA. Chemicals for antioxidant assays were acquired from Fujifilm Wako Pure Chemical Corporation, Osaka, Japan. Elastase from porcine pancreas Type IV, tyrosinase from mushroom lyophilized powder, N-Succinyl-Ala-Ala-Ala-p-nitroanilide (SANA), L-tyrosine, oleanolic acid, vanillin, myricetin, and all buffer components were acquired from Sigma-Aldrich, St. Louis, MO, USA.

Pure momilactones A, B, and tricin were isolated previously from rice husk by our laboratory [12]. Briefly, the ethyl acetate (EtOAc) extract of 7 kg Koshihikari husks was separated by open column chromatography over silica gel with the mobile phase as combinations of hexane and EtOAc. Momilactones A and B were isolated from the eluant hexane:EtOAc (8:2) by a repeated column chromatography while tricin (a yellow powder) was purified from the last fractions of the eluant hexane: EtOAc (7:3). The identification of such pure compounds is described in detail in the following parts.

### 2.2. Rice Grains and Sample Preparations

Grains of five rice varieties were investigated in this study, of which, four varieties were refined type and one brown rice. All varieties are the most commercially popular rice in Japan. The information of rice samples is shown in Table 1, in which, all grains were purchased from Japan Agricultural Cooperatives (JA), Hiroshima, Japan.

The extraction of rice grains was done followed methods described in previous studies [12,14]. In brief, except for the cooked Koshihikari sample, dried and powdered rice grains (200 g) were extracted with 300 mL of methanol for 1 week. After separating from hexane extract, the methanolic phase was filtered and dried by a vacuum evaporator (Rotavapor^®^ R-300, Nihon Buchi K.K., Tokyo, Japan). The residue was then blended with 50% aqueous methanol. After shaking, the mixture was loaded into a Sep-Pak^®^ Plus C18 cartridge (Waters Cooperation, Milford, MA, USA). Subsequently, the cartridge was pre-washed with 2 mL of 50% methanol and eluted with 10 mL of pure methanol. Eventually, the methanolic elutions were combined and adjusted into the concentration of 10 mg/mL and stored in a fridge (4 °C) for further measurements. In the case of cooked Koshihikari sample, rice was cooked by a common electric cooker. After cooking, rice was dried by a convection oven under 50 °C for 1 week to yield 65 g dry weight prior to being extracted by the above protocol.

### 2.3. Quantification and Confirmation of MA, MB, and Tricin in Rice Grains

#### 2.3.1. Quantification of MA and MB in Rice Grains by UPLC-ESI-MS

Standard MA and MB were authenticated by reliable spectroscopic methods in previous studies [12,13,14]. The UPLC-ESI-MS system consisted of an LTQ Orbitrap XL mass spectrometer (Thermo Fisher Scientific, CA, USA) equipped with a source of electrospray ionization (ESI). The liquid chromatography (LC) was carried out by injecting 3.0 µL of a sample (in methanol) into the Acquity UPLC^®^ BEH C18 (1.7 µm, 50 × 2.1 mm i.d.) column (Waters Cooperation, Milford, MA, USA). The column temperature was maintained at 25 °C. The chromatography was run in a gradient mode with a flow rate of 300 µL/min. The gradient of mobile phase was established as follows: 50% solvent A (0.1% trifluoroacetic acid in water) and 50% solvent B (0.1% trifluoroacetic acid in acetonitrile) over 0–5 min, then increased to 100% B over 5–10 min which was upheld for 0.1 min, finally the column was equilibrated by the initial condition for 5 min. The total operation time was 15.1 min. The ESI condition and MS model was set up similar to the previous study [12,13]. The presence of MA and MB in rice grains was confirmed by comparing their extracted ion chromatograms (EIC) and mass spectra of samples with those of standard momilactones. The area of a peak that matched with the retention time of either MA or MB in the EIC was used to calculate the amount of each compound by a linear model. The calibration curves of MA (y = 4577x − 811557, *r*^2^ = 0.9714) and MB (y = 2679x − 328396; *r*^2^ = 0.9952) established from 10 to 10000 ng/mL were employed for estimation of MA and MB contents in rice grains. Detection and quantitation limits were 0.68 and 2.05 ng/mL for MA and 0.27 and 0.83 ng/mL for MB.

#### 2.3.2. Identification and Quantification of Tricin by ESI-MS and HPLC

The isolated tricin was confirmed by flow injection analysis (FIA) coupled with ESI-MS (FIA-ESI-MS) as described above. At first, tricin was dissolved in a mixture of methanol and acetonitrile (8:2, *v/v*) and injected to ESI system (positive ion mode) by an auto-sampler with a flow rate of 200 µL/min. The injection volume was 1 µL. The ESI condition was set up as follows: ion source voltage was 4.5 kV, capillary voltage was 50 V, tube lens offset was 80 V; capillary temperature was 330 °C, vaporizer temperature was −65 °C; nitrogen was the gas carrier with the sheath flow rate was 50 arb, and the aux flow rate was 10 arb. The mass spectra were recorded at a resolution of 60000 with a scan range of 100–2000 mass-to-charge (*m/z*). The full-scan and data-dependent scan spectra for MS/MS on [M + H]^+^ were acquired and processed by using Xcalibur software integrated with the NIST database. The online database (Pubchem, National Center for Biotechnology Information, U.S. National Library of Medicine, Bethesda MD, USA) and literature [15,16] were used for reference to MS/MS spectra.

The amount of tricin in rice grain samples was quantified by the HPLC system which included PU-4180 RHPLC pump, LC-Net II/ADC controller, and UV-4075 UV/Vis detector (Jasco, Tokyo, Japan). The stationary phase was XBridge BEH Shield RP18 (130Å, 5 µm, 2.1 × 100 mm) column (Waters Cooperation, Milford, MA, USA). The mobile phase consisted of solution A (0.1% aqueous formic acid) and solution B (100% acetonitrile), which was adjusted in a gradient program as follows: 5% B during 0–2 min, a linear increase from 5 to 70% B during 2–12 min, 100% B from 12–16 min and maintained for 6 min, 100% B to 5% during 22–24 min and other 10 min for equilibration. The injection volume was 3 µL and the flow rate was 400 µL/min. The operation was carried out in 35 min under room temperature (24–26 °C). The corresponding peak from a sample was detected at 350 nm and its area was used to calculate the amount (µg per g dry weight, µg/g DW) over the standard curve built from the isolated tricin (from 1 to 100 µg/mL, y = 23972x − 9463, *r*^2^ = 0.9999). The limits of detection and quantitation were 0.04 ng/mL and 0.12 ng/mL, respectively.

### 2.4. In Vitro Antioxidant and Elastase and Tyrosinase Inhibitory Assays

All biological activities of isolated compounds were assayed under the in vitro condition by using a microplate reader (Multiskan^TM^ Microplate Spectrophotometer, Thermo Fisher Scientific, Osaka, Japan) and U-shape microplates (Greiner Bio-one, Monroe, NC, USA).

#### 2.4.1. Antioxidant Activities

The antioxidant capacities of MA, MB, and the mixture of MA and MB (MAB, 1:1, *v/v*) were evaluated and compared with tricin by 2,2’-azino-bis radical cation (ABTS•+) decolorization, reducing power, and β-carotene bleaching methods which were described previously by Quan et al. [17].

#### 2.4.2. Elastase and Tyrosinase Inhibitory Activities

Elastase inhibition was assayed basing the method reported by Tu and Tawata [4] with minor refitted changes. Particularly, 200 μL of the buffer substrate (1 mM SANA 0.1 M Tris-HCl buffer, pH 8.0) was added to 20 μL of a stock sample (in methanol) in a well of the 96-well plate. The solutions were vortexed and incubated for 5 min at 25 °C, and then 15 μL of pancreatic elastase (0.1 U/mL) was added. After mixing, the microplate was incubated at 25 °C for 12 min and subsequently, the absorbance was measured at 410 nm. Negative controls were performed with methanol, while oleanolic acid was used as a positive control.

The inhibition of tyrosinase was determined by the protocol of Chompoo et al. [1] with a minor alteration. Briefly, in each well of a 96-well plate, 20 μL of sample were mixed with 120 μL of phosphate buffer (20 mM, pH 6.8) and 20 μL of tyrosinase (500 U/mL in buffer), respectively. After five minutes-incubation at 25 °C, an adequate amount of 50 μL L-tyrosine substrate (2 mM in deionized water) was pipetted to start the reaction. The mixture was slightly shaken and incubated for other 10 min at 25 °C prior to be measured under 470 nm. Myricetin and vanillin were used as positive references, whereas methanol was the negative control.

The inhibition percentage of enzymatic assays was calculated as

Inhibition (%) = (1 − (C − D)/(A − B)) × 100(1)

where A is the absorbance of the control with the enzyme, B is the absorbance of the control without the enzyme, C is the absorbance of the sample with the enzyme, and D is the absorbance of the sample without the enzyme. The IC_50_ value was defined as the concentration of a sample that suppressed 50% of the enzymatic reaction over the negative control.

### 2.5. Statistical Analysis

Data were elaborated on the Minitab 16.0 software (Minitab Inc., State College, PA, USA). All assays were thrice implemented, and results were displayed as means ± standard errors (SE). IC_50_ values were obtained from a dose–response curve between sample concentrations and percent inhibitions. Pearson’s correlation was established to analyze the relationships among biological activities of MA and MB. Significant differences among tests were determined by one-way ANOVA at *p* < 0.05.

## 3. Results

### 3.1. Confirmation of MA, MB, and Tricin

Isolated MA and MB were confirmed and identified by previous reports [12,13,14]. By UPLC-ESI-MS, extracted ion chromatograms showed a good separation of MA and MB, in which, MA was detected at 3.78 min with a major ion *m/z* 315 and MB was eluted at 2.33 min with a major ion *m/z* 331, see Supplementary data, Figures S1 and S2.

**Figure 1:**
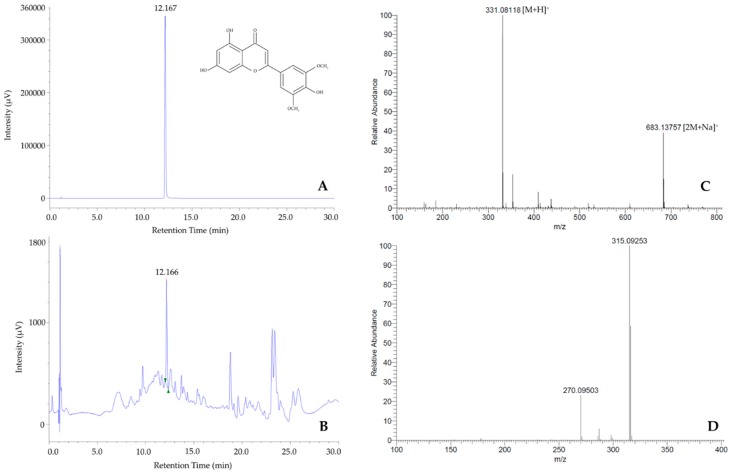
HPLC chromatograms of isolated tricin (**A**) and detected tricin in rice grain extract (**B**); Mass spectrum (**C**) and MS-MS product ions for *m/z* 331 of isolated tricin (**D**) by FIA-ESI.

The purity of isolated tricin is performed by HPLC chromatogram (Figure 1A), of which, one peak was detected at 12.167 min. The existence of tricin in rice grain extracts was determined by comparing retention time of a peak that coincident with the standard tricin (Figure 1B), which was also confirmed by measuring the mixture of standard tricin and extract. By FIA-ESI-MS analysis, mass spectrum shows two major ions including [M + H]^+^ at *m/z* 331 and [2M + Na]^+^ at *m/z* 683, see Figure 1C. Besides, MS-MS for *m/z* 331 presenting two major product ions at 315 and 270 (Figure 1D) which is entirely similar to the reported tricin from mass databases and literature [15,16].

### 3.2. Antioxidant Activities

**Figure 2:**
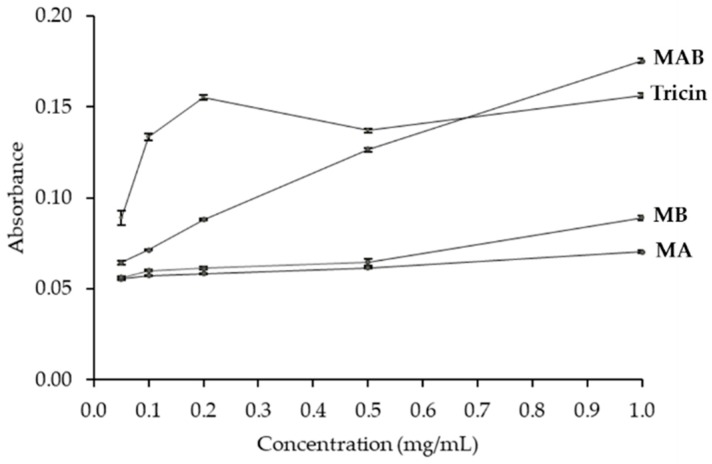
Reducing power activity of momilactones A, B, and tricin. Data expressed as means ± standard errors. MA, momilactone A; MB, momilactone B; MAB, the mixture of MA and MB at 1:1, *v/v*.

Antioxidant properties of MA, MB, and MAB are displayed in Table 2 and Figure 2. According to the result of ABTS assay, the individual MA and MB present a weaker activity with IC_50_ values of 2.8 and 1.3 mg/mL, respectively than that of the standard antioxidant, butylated hydroxytoluene (BHT, IC_50_ = 0.080 mg/mL). However, the mixture of MA and MB (MAB, 1:1, *v/v*) exhibits a synergistic activity (IC_50_ = 0.3 mg/mL) which is significantly higher than activities of MA and MB individually and was in line with the antioxidant ability of tricin (IC_50_ = 0.3 mg/mL). Notably, by β-carotene bleaching assay, MA, MAB, and tricin performed stronger lipid peroxidation inhibitions (% LPI = 75.2, 80.0, and 76.1, respectively) than MB did (% LPI = 61.7) at the same concentration of 1 mg/mL. By this assay, antioxidant activities of MA, MAB, and tricin were determined to be close that of the standard BHT (%LPI = 86.7).

Although all samples presented a negligible antioxidant ability in terms of reducing power assay, the result showed that the mixture MAB and tricin were significantly powerful than either MA or MB (Figure 2). Except for 1 mg/mL which MB exerted stronger potential than MA, the levels of antioxidant activity between MA and MB were negligible (Figure 2).

### 3.3. Potential Anti-Elastase and Anti-Tyrosinase Activities of Momilactones A and B

By *in vitro* assays, momilactones A and B were remarkably active in the inhibition of two key enzymes elastase and tyrosinase which related to wrinkles and freckles (Table 3). At a concentration of 2 mg/mL, MA inhibited 30.9 and 37.6% of pancreatic elastase and tyrosinase activities, respectively, meanwhile, MB suppressed only 18.5 and 12.6% of reactions, respectively. The inhibitory effect of MAB (32.0%) mixture was in line with that of MA in the case of elastase assay. On the other hand, the standard oleanolic acid caused a 50% inhibition on pancreatic elastase at 0.3 mg/mL, whereas myricetin had an IC_50_ value of 0.74 mg/mL for tyrosinase suppression.

Remarkably, at a concentration of 2 mg/mL, MA, MAB, and MB showed a significantly stronger anti-elastase activity than tricin. Additionally, the anti-tyrosinase activity of MA and MAB was significantly higher than that of vanillin (13.1%) and tricin (15.7%) which were reported as potent tyrosinase inhibitors [10,18]. Meanwhile, MB’s activity (12.6%) was comparable with vanillin.

### 3.4. Momilactones A, B, and Tricin Contents in Rice Grains

The quantities of MA, MB, and tricin in various common rice grains are shown in Table 4. It is clear that the brown rice grain (KT4) contains a much higher content of active metabolites (MA, MB, and tricin) than refined rice grains. In particular, MA and MB were determined at the highest amount (1.56 and 1.61 µg/g DW, respectively) in the KT4 variety. Among refined grains, Koshihikari grain accommodated the highest amount of MA (0.46 µg/g DW) and MB (0.41 µg/g DW). Notably, both MA and MB contents (0.09 and 0.08 µg/g DW, respectively) in cooked Koshihikari rice reduced approximately 5-fold compared to those of the uncooked grain (Table 4).

In terms of tricin content, the highest amount was quantified in Koshihikari refined grain (0.55 µg/g DW) whereas the lowest one was in KT1 (0.03 µg/g DW). The tricin content of cooked Koshihikari was decreased a half (0.23 µg/g DW) compared to that of the uncooked grain.

## 4. Discussion

Momilactones A and B are important compounds in the defense-mechanism pathway of rice (*Oryza sativa*). Recently, we have revealed the potential antidiabetic and anti-obesity activities of MA and MB via in vitro enzymatic assays. In this study, we focused on the novel biological activity (anti-skin-aging) of these active metabolites and compared their capacity with known beneficial compounds in rice. In fact, Fukuta et al. [19] reported the antioxidant activity of MA and MB, however, the study only showed a weak activity on 2,2-diphenyl-1-picrylhydrazyl (DPPH) reagent, by which, the half maximal effective concentrations (EC_50_) for MA and MB were 783.9 and 790.7 µg, respectively [19]. In the present study, by ABTS, reducing power and β-carotene bleaching methods, we successfully evaluated the antioxidant capacity of MA and MB. For the first time, the mixture of MA and MB (MAB, 1:1, *v/v*) was found to be more significantly potent than the single MA and MB in terms of ABTS and reducing power assays. Though the anti-ABTS radical activity of MA and MB was compared to that of BHT (IC_50_ = 0.080 mg/mL), a strong known antioxidant, further equivalent antioxidant capacity assays should be conducted using other reference standards such as 6-hydroxy-2,5,7,8-tetramethylchroman-2-carboxylic acid (Trolox), ascorbic acid, and butylated hydroxyanisole (BHA) to gauge the anti-radical capacity of MA and MB more accurately. In fact, the accretion of free radicals induced by UV exposure can provoke skin problems via a series of cell destruction mechanisms [20]. These dermatological disturbances, including skin elasticity decrease and hyperpigmentation, were reported to be clinically correlated with ROS [21]. Therefore, the discovery of ABTS free radical scavengers MA and MB could be useful for the prospective development of anti-skin aging drugs.

Interestingly, this study indicated the first time MA possessing stronger activities than MB by β-carotene bleaching and elastase and tyrosinase inhibitory activities. Prominently, MA and MB performed higher anti-tyrosinase activities than vanillin and tricin which were affirmed as potential tyrosinase inhibitors [10,18]. The Pearson’s correlation results (Table 5) showed a significant positive linear correlation between lipid peroxidation and elastase inhibitory activities of MA and MB with a coefficient *r*^2^ = 0.944 at *p* < 0.05. Meanwhile, the correlation between tyrosinase assay and either β-carotene bleaching, or elastase assay was not significant as *p* > 0.05. The results suggested that the lipid peroxidation inhibitory effect might determine the anti-elastase activity of MA and MB herein. On the other consideration, Mu et al. [10] reported that tricin was a potent inhibitor that could inhibit 50% of tyrosinase activity at a concentration of 0.3 mg/mL [10]. In the present study, isolated tricin from rice husk provided only 15.7% inhibition at a concentration of 2 mg/mL. In addition, tricin was confirmed as a non-competitive inhibitor of tyrosinase [10]; therefore, further biological models should be tested to scrutinize the potential impacts on tyrosinase inhibitory action mode of tricin. Furthermore, vanillin and tricin were reported as dominant compounds in various parts of rice [22,23]. Rice and its products were investigated as outstanding sources for the treatment of skin diseases as well [24,25]. Among identified cosmeceutical compounds from rice, flavonoids [26], ferulic acid, γ-oryzanol, and phytic acid [27] were the most common. Also, previous studies signified the cosmeceutical property of the complex terpene-lactone compounds such as 2,3-epoxyjuanislamin [28], ginkgolide B [29], and α-cyclocostunolide [30] which potently inhibited the melanogenesis. Therefore, findings of the present study introduced new members of terpene-lactone (MA and MB) to the anti-aging substance class. The inhibitory assays on elastase and tyrosinase activities showed that MA and MB can be promising candidates of skin-stretching and skin-whitening materials. However, the applicability of these compounds should be examined more thoroughly, focusing on skin penetration, skin irritation, and other adverse effects by various in vivo models and pre-clinical trials.

To date, momilactones A and B have been recognized to have valuable effects on human health comprising antidiabetic [12], anti-obesity [13], anticancer [31,32,33], and antioxidant and antiaging properties. Momilactones A and B have been quantified by various techniques but mostly by HPLC [12,13,34]. In previous studies, we found that the sensitivity of MA and MB quantifications by HPLC could be enhanced by applying the SEP-PAK C18 cartridge in preparation of the rice husk sample [14]. The same application of the C18 cartridge was also reported by [35]. Therefore, in the present study, we investigated the MA and MB contents in rice grains of various commercial varieties by using SEP-PAK C18 cartridge purification integrated with an advanced UPLC-ESI-MS. The profile of MA and MB contents in various rice grains provided by this study may contribute an important role to future food, medicinal, and cosmetic industries. On the other consideration, tricin, an important flavonoid widely distributed in herbaceous plants, has been gained more and more attention recently due to its pharmaceutical potentials and validated safety [36,37]. Tricin has been affirmed to possess many valuable medicinal properties including antioxidant, antiaging, anticancer, and cardioprotective activities [38]. In rice, tricin is reported to be present in bran [37], stem, leaf [39], and hull [15,40]; however, there have been few studies quantifying tricin in rice grains, especially in refined grains. In the current investigation, the cooking process caused a significant reduction of MA, MB, and tricin contents in Koshihikari rice grain. However, the revelation of such bioactive compounds in the cooked rice strengthens the hypothesis that eating a certain amount of rice daily may prevent aging and several diseases [12]. Science has proven that proper nutrition and physical exercise are more important in disease prevention [41]. Thus, the development of medicinal cereals is an up-and-coming direction in the study of drug improvement associated with nutrient diets from natural origins. Nevertheless, the digestion process of MA, MB, and tricin should be more elaborated by in vivo or animal models to assess the real absorption as well as the alleviation of such compounds through the digestive system.

## 5. Conclusions

This study is the first observation of antioxidant and anti-skin-aging properties of MA and MB. The biological activities of MA and MB were assayed by in vitro modes and compared with tricin, a potent antioxidant, antiaging, and anticancer molecule. Quantities of MA, MB, and tricin were varied among Japanese rice cultivars. The use of liquid chromatography-electrospray ionization-mass spectrometry (UPLC-ESI-MS) and HPLC is effective to identify MA, MB, and tricin in rice grains. Although MA, MB, the mixture MAB and tricin are potential inhibitors for skin aging treatments, the level of inhibitory activity of MA was stronger than either MB or tricin. The three compounds MA, MB, and tricin in rice grain are promising for development of supplements and cosmetics for skin protection and antioxidants.

## Figures and Tables

**Table 1:** Information of rice grains.

Code	Name of Rice Grain	Type	Origin
Ko	Koshihikari	Refined	Japan
CoKo	Koshihikari (cooked rice)	Refined	Japan
KT1	Shinnosuke	Refined	Japan
KT2	Seiten no hekireki	Refined	Japan
KT3	Ginga no shizuku	Refined	Japan
KT4	Ho no mai	Brown	Japan

**Table 2:** Antioxidant activities of momilactones A, B, and tricin in terms of 2,2’-azino-bis (ABTS) and β-carotene bleaching assays.

Sample	IC_50_ value of ABTS assay (mg/mL)	β-Carotene bleaching assay at a concentration of 1 mg/mL (% LPI)
MA	2.838 ± 0.010 d	75.234 ± 0.855 b
MB	1.283 ± 0.002 c	61.690 ± 1.640 c
MAB	0.319 ± 0.002 b	79.990 ± 1.080 b
Tricin	0.312 ± 0.006 b	76.070 ± 2.050 b
BHT	0.080 ± 0.001 a	86.667 ± 0.327 a

Data presented as means ± standard errors (*n* = 3). The antioxidant strength is represented following the alphabetic order (a–d). Lower IC_50_ values indicate stronger antioxidant activities. Means within a same column followed by similar letters are not significantly different by Turkey’s test (*p* < 0.05). MA, momilactone A; MB, momilactone B; MAB, the mixture of MA and MB at 1:1, *v/v*; BHT, butylated hydroxytoluene; LPI, lipid peroxidation inhibition.

**Table 3:** Inhibitory activities on pancreatic elastase and tyrosinase of momilactones A, B, and tricin at 2 mg/mL.

Sample	Inhibition percentage (%)	
	Pancreatic elastase	Tyrosinase
MA	30.863 ± 0.267 a	37.590 ± 0.269 a
MB	18.504 ± 0.561 b	12.600 ± 0.521 d
MAB	32.032 ± 0.472 a	19.714 ± 0.517 b
Tricin	13.973 ± 0.460 c	15.692 ± 0.525 c
Vanillin	-	13.124 ± 0.276 d
Oleanolic acid (IC_50_)	0.277 ± 1.100 mg/mL	-
Myricetin (IC_50_)	-	0.736 ± 0.006 mg/mL

Data presented as means ± standard errors (*n* = 3). The enzymatic inhibition is represented following the alphabetic order (a–d). Means within a same column followed by similar letters are not significantly different by Turkey’s test (*p* < 0.05). MA, momilactone A; MB, momilactone B; MAB, the mixture of MA and MB (1:1, *v/v*); -, not determined.

**Table 4:** Momilactones A, B, and tricin contents (µg/g DW) in various rice grains.

Code	Type	Momilactones		Tricin
		MA	MB	
Ko	Refined	0.46 c	0.41 b	0.55 a
CoKo	Refined	0.09 ef	0.08 de	0.23 c
KT1	Refined	0.05 fg	0.05 ef	0.03 j
KT2	Refined	0.08 efg	0.07 de	0.13 e
KT3	Refined	0.13 de	0.15 c	0.09 g
KT4	Brown	1.56 a	1.61 a	0.24 b

Results are presented in means (*n* = 3); Ko, Koshihikari; CoKo, cooked Koshihikari, KT1, shinnosuke rice; KT2, seiten no hekireki rice; KT3, ginga no shizuku rice; KT4, ho no mai. Means within a same column followed by similar letters are not significantly different by Turkey’s test (*p* < 0.05).

**Table 5:** Pearson’s correlation among β-carotene bleaching, elastase, and tyrosinase inhibitory activities of MA and MB.

Activity	β-Carotene	Elastase
**Elastase**	0.944	1.000
	0.000	
**Tyrosinase**	0.512	0.658
	0.159	0.054

In a cell, the upper value is correlation coefficient (*r*^2^) while the lower one is *p*-value.

## Supplementary Materials

The following are available online at https://www.mdpi.com/2304-8158/8/12/602/s1, Figure S1: Extracted ion chromatography and mass spectrum of standard momilactones A (MA) and B (MB); Figure S2: Extracted ion chromatograms (EIC) and mass spectra (MS) of MA and MB in rice grain extracts by UPLC-ESI-MS.
