# Supplementary material for: Momilactones A, B, and Tricin in Rice Grain and By-Products are Potential Skin Aging Inhibitors
Source: Foods. 2019 Nov 21;8(12):602. doi: 10.3390/foods8120602 (PMC6963690; doi:10.3390/foods8120602)
Supplement: Supplementary file 1 [file foods-08-00602-s001.pdf]

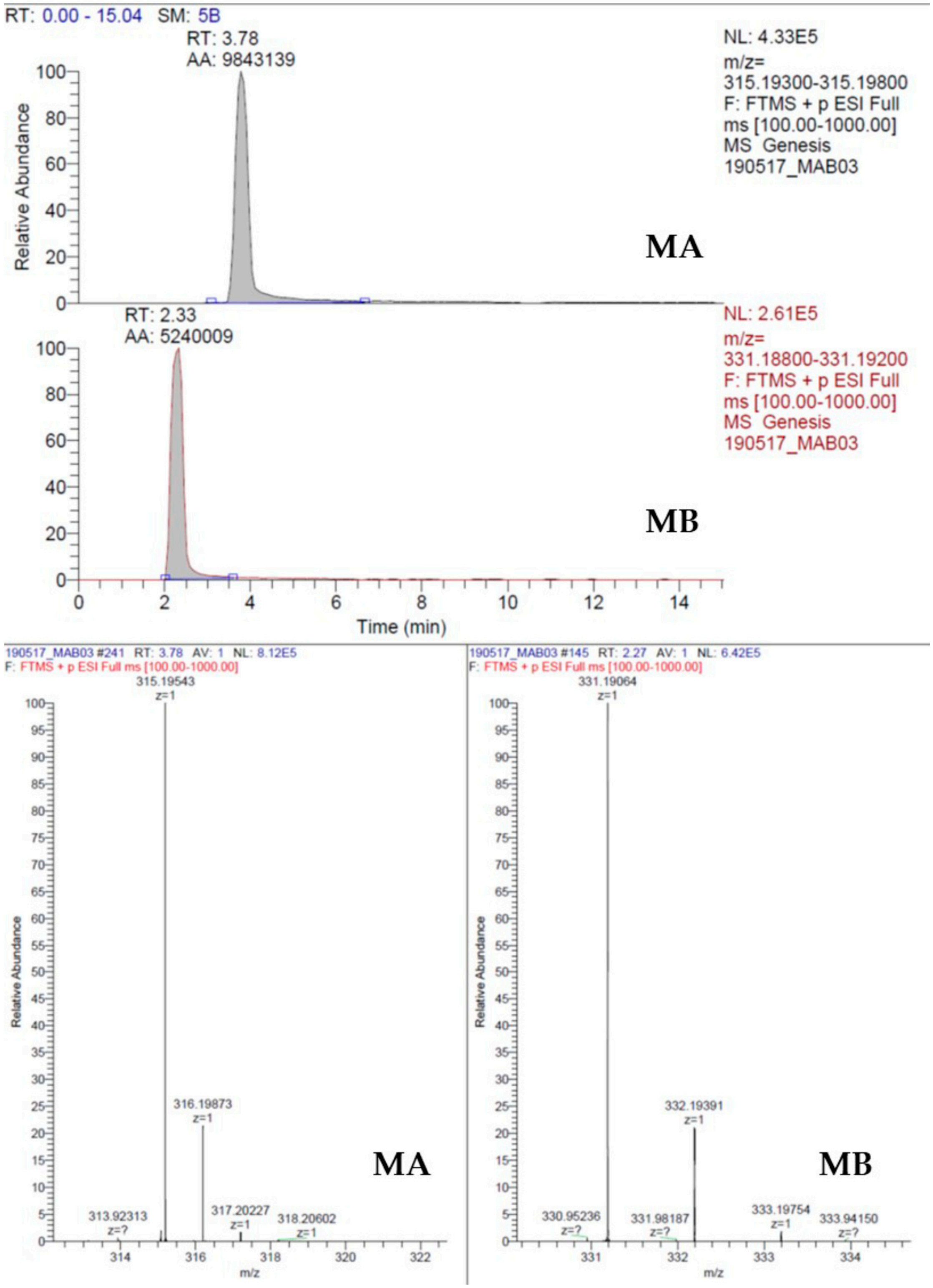


**Figure S1**. Extracted ion chromatography and mass spectrum of standard momilactones A (**MA**) and B (**MB**)

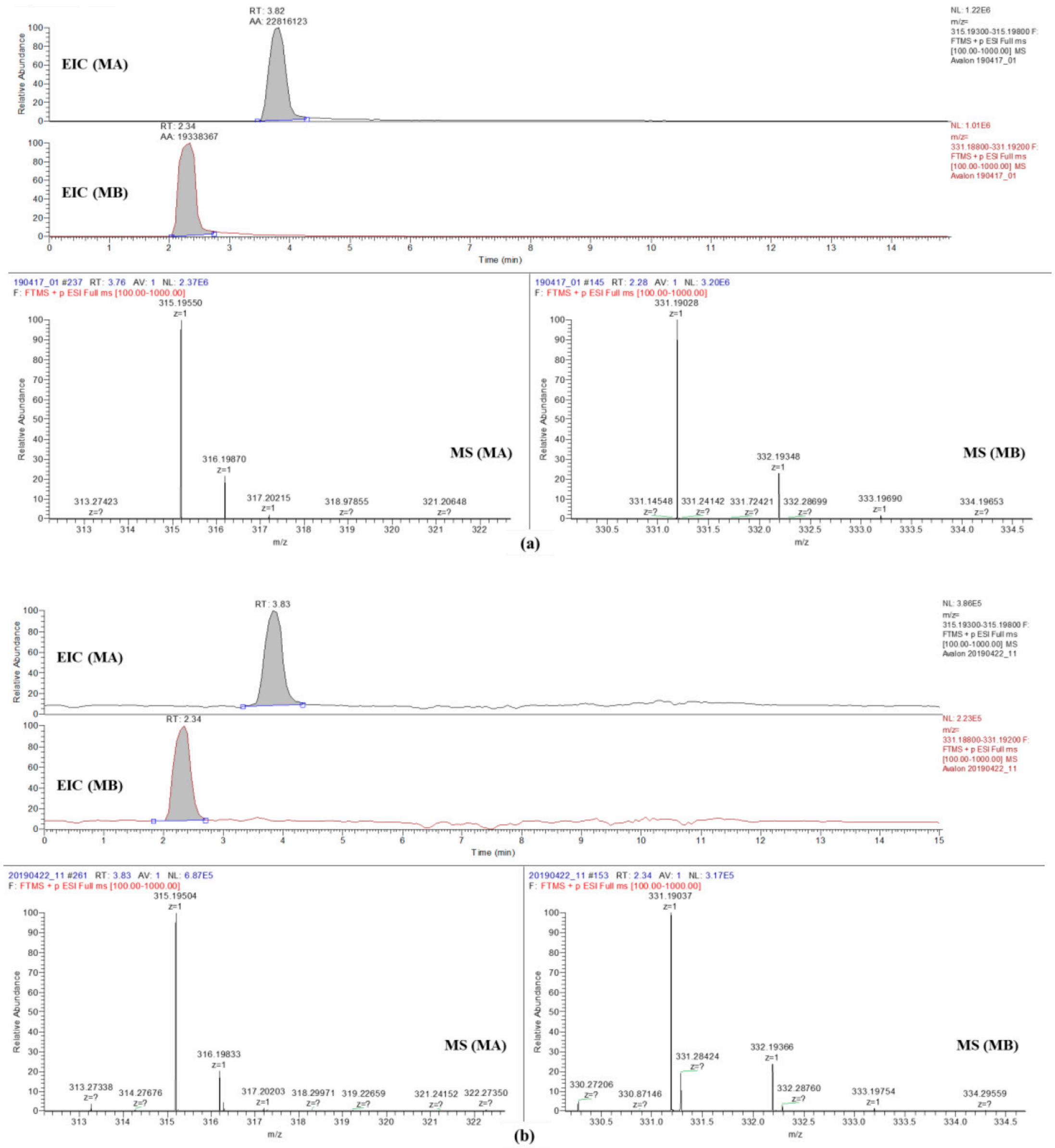
RT: 3.82
AA: 22816123
EIC (MA)
NL: 1.22E6
m/z= 315.19300-315.19800 F: FTMS + p ESI Full ms [100.00-1000.00] MS Avalon 190417_01
RT: 2.34
AA: 19338367
EIC (MB)
NL: 1.01E6
m/z= 331.18800-331.19200 F: FTMS + p ESI Full ms [100.00-1000.00] MS Avalon 190417_01
Relative Abundance
Time (min)
190417_01 #237 RT: 3.76 AV: 1 NL: 2.37E6
F: FTMS + p ESI Full ms [100.00-1000.00]
315.19550 z=1
316.19870 z=1
313.27423 z=?
317.20215 z=1
318.97855 z=?
321.20648 z=?
MS (MA)
m/z
190417_01 #145 RT: 2.28 AV: 1 NL: 3.20E6
F: FTMS + p ESI Full ms [100.00-1000.00]
331.19028 z=1
332.19348 z=1
331.14548 z=?
331.24142 z=?
331.72421 z=?
332.28699 z=?
333.19690 z=1
334.19653 z=?
MS (MB)
(a)
RT: 3.83
EIC (MA)
NL: 3.86E5
m/z= 315.19300-315.19800 F: FTMS + p ESI Full ms [100.00-1000.00] MS Avalon 20190422_11
RT: 2.34
EIC (MB)
NL: 2.23E5
m/z= 331.18800-331.19200 F: FTMS + p ESI Full ms [100.00-1000.00] MS Avalon 20190422_11
20190422_11 #261 RT: 3.83 AV: 1 NL: 6.87E5
F: FTMS + p ESI Full ms [100.00-1000.00]
315.19504 z=1
316.19833 z=1
313.27338 z=?
314.27676 z=?
317.20203 z=1
318.29971 z=?
319.22659 z=?
321.24152 z=?
322.27350 z=?
MS (MA)
20190422_11 #153 RT: 2.34 AV: 1 NL: 3.17E5
F: FTMS + p ESI Full ms [100.00-1000.00]
331.19037 z=1
331.28424 z=?
332.19366 z=1
330.27206 z=?
330.87146 z=?
332.28760 z=?
333.19754 z=1
334.29559 z=?
MS (MB)
(b)

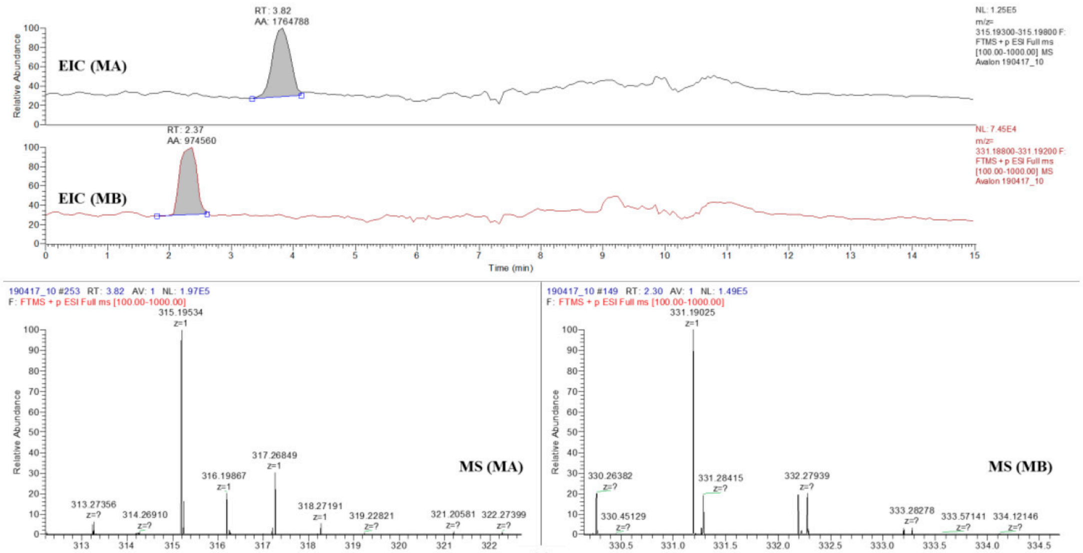
RT: 3.82
AA: 1764788
EIC (MA)
RT: 2.37
AA: 974560
EIC (MB)
Relative Abundance
Time (min)
NL: 1.25E5
NL: 7.45E4
190417_10 #253 RT: 3.82 AV: 1 NL: 1.97E5
F: FTMS + p ESI Full ms [100.00-1000.00]
315.19534
MS (MA)
m/z
190417_10 #149 RT: 2.30 AV: 1 NL: 1.49E5
F: FTMS + p ESI Full ms [100.00-1000.00]
331.19025
MS (MB)

(c)

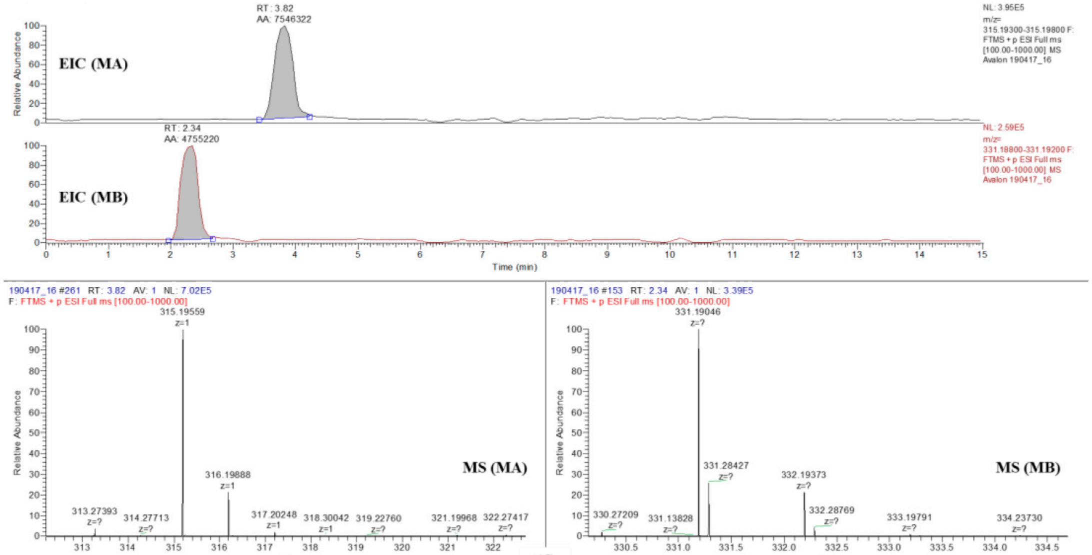
RT: 3.82
AA: 7546322
EIC (MA)
RT: 2.34
AA: 4755220
EIC (MB)
Relative Abundance
Time (min)
NL: 3.95E5
NL: 2.59E5
190417_16 #261 RT: 3.82 AV: 1 NL: 7.02E5
F: FTMS + p ESI Full ms [100.00-1000.00]
315.19559
MS (MA)
m/z
190417_16 #153 RT: 2.34 AV: 1 NL: 3.39E5
F: FTMS + p ESI Full ms [100.00-1000.00]
331.19046
MS (MB)

(d)

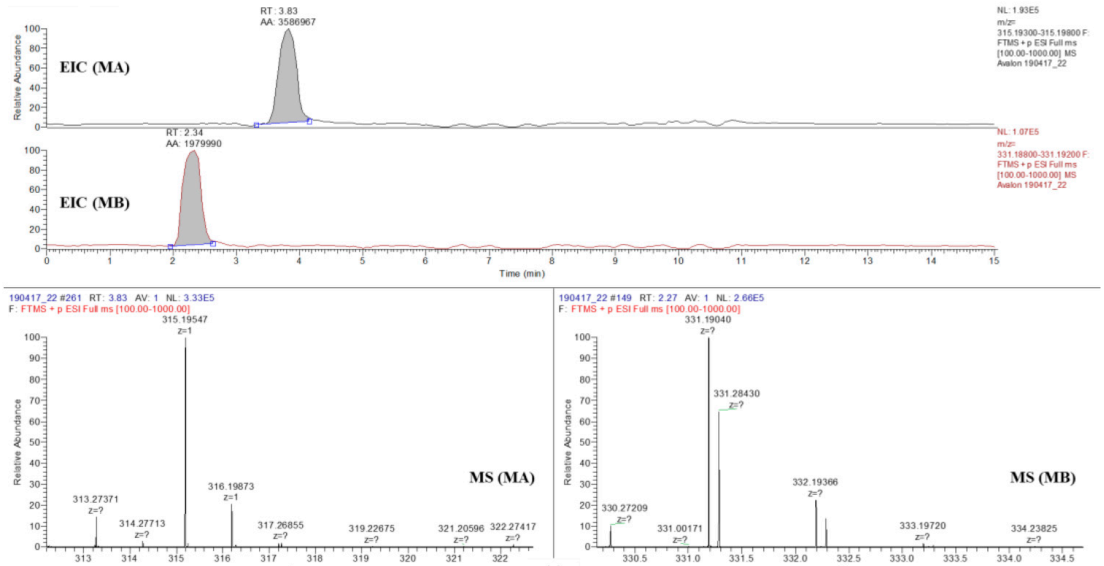
RT: 3.83
AA: 3586967
EIC (MA)
RT: 2.34
AA: 1979990
EIC (MB)
Relative Abundance
Time (min)
NL: 1.93E5
m/z= 315.19300-315.19800 F: FTMS + p ESI Full ms [100.00-1000.00] MS Avalon 190417_22
NL: 1.07E5
m/z= 331.18800-331.19200 F: FTMS + p ESI Full ms [100.00-1000.00] MS Avalon 190417_22
190417_22 #261 RT: 3.83 AV: 1 NL: 3.33E5
F: FTMS + p ESI Full ms [100.00-1000.00]
315.19547
z=1
316.19873
z=1
313.27371
z=?
314.27713
z=?
317.26855
z=?
319.22675
z=?
321.20596
z=?
322.27417
z=?
MS (MA)
m/z
190417_22 #149 RT: 2.27 AV: 1 NL: 2.66E5
F: FTMS + p ESI Full ms [100.00-1000.00]
331.19040
z=?
331.28430
z=?
332.19366
z=?
330.27209
z=?
331.00171
z=?
333.19720
z=?
334.23825
z=?
MS (MB)
m/z

(e)

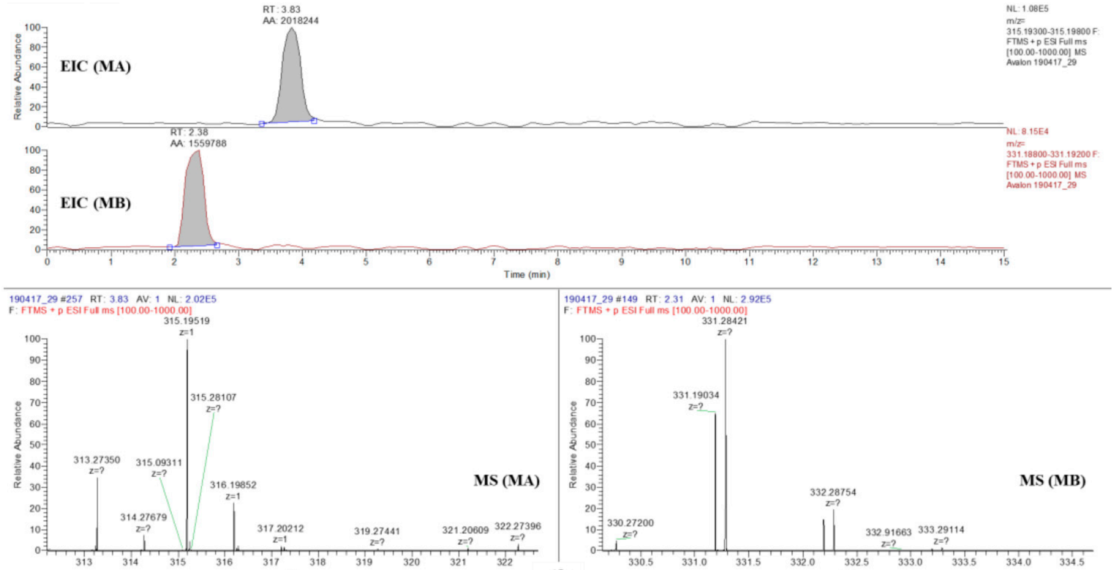
RT: 3.83
AA: 2018244
EIC (MA)
RT: 2.38
AA: 1559788
EIC (MB)
Relative Abundance
Time (min)
NL: 1.08E5
m/z= 315.19300-315.19800 F: FTMS + p ESI Full ms [100.00-1000.00] MS Avalon 190417_29
NL: 8.15E4
m/z= 331.18800-331.19200 F: FTMS + p ESI Full ms [100.00-1000.00] MS Avalon 190417_29
190417_29 #257 RT: 3.83 AV: 1 NL: 2.02E5
F: FTMS + p ESI Full ms [100.00-1000.00]
315.19519
z=1
315.28107
z=?
313.27350
z=?
315.09311
z=?
316.19852
z=1
314.27679
z=?
317.20212
z=1
319.27441
z=?
321.20609
z=?
322.27396
z=?
MS (MA)
m/z
190417_29 #149 RT: 2.31 AV: 1 NL: 2.92E5
F: FTMS + p ESI Full ms [100.00-1000.00]
331.28421
z=?
331.19034
z=?
332.28754
z=?
330.27200
z=?
332.91663
z=?
333.29114
z=?
MS (MB)
m/z

(f)

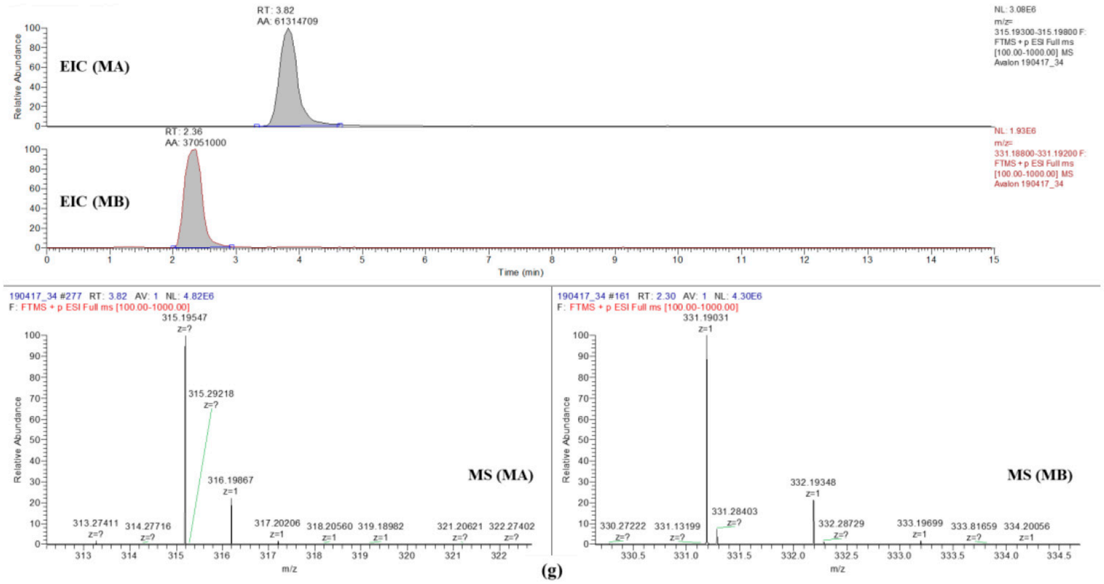
EIC (MA)
EIC (MB)
MS (MA)
MS (MB)
Time (min)
m/z
Relative Abundance

(g)

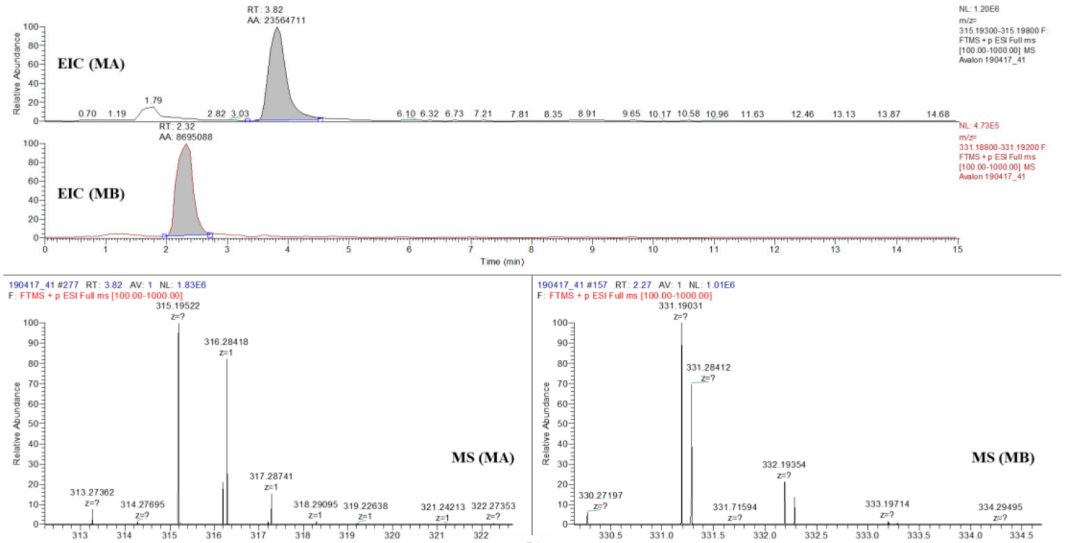
EIC (MA)
EIC (MB)
MS (MA)
MS (MB)
Time (min)
m/z
Relative Abundance

(h)

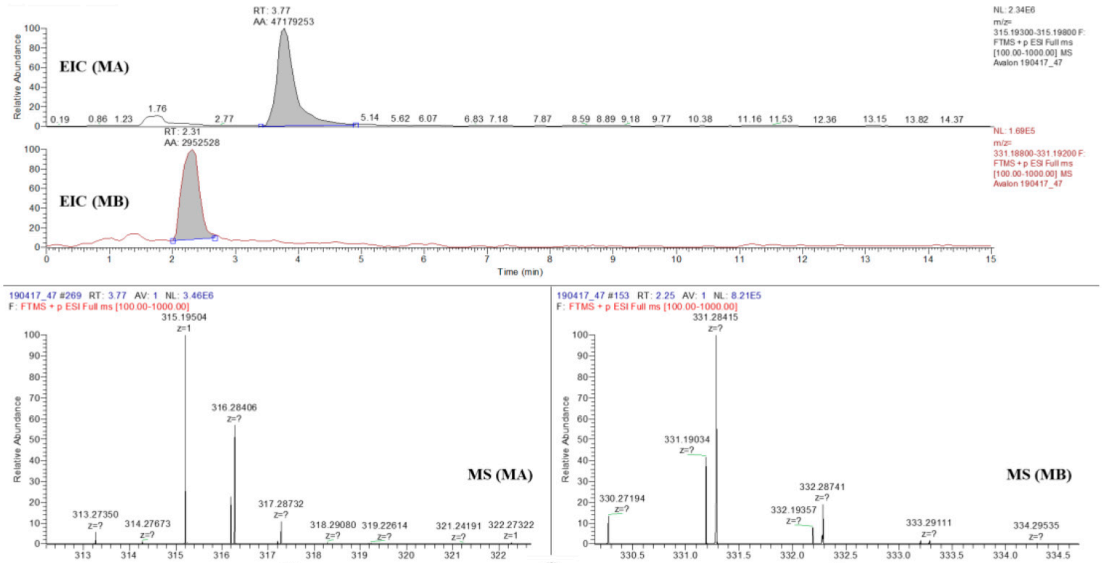
EIC (MA)
RT: 3.77
AA: 47179253
NL: 2.34E6
m/z= 315.19300-315.19800 F: FTMS + p ESI Full ms [100.00-1000.00] MS Avalon 190417_47
EIC (MB)
RT: 2.31
AA: 2952528
NL: 1.69E5
m/z= 331.18800-331.19200 F: FTMS + p ESI Full ms [100.00-1000.00] MS Avalon 190417_47
Relative Abundance
Time (min)
190417_47 #269 RT: 3.77 AV: 1 NL: 3.46E6
F: FTMS + p ESI Full ms [100.00-1000.00]
315.19504
316.28406
MS (MA)
190417_47 #153 RT: 2.25 AV: 1 NL: 8.21E5
F: FTMS + p ESI Full ms [100.00-1000.00]
331.28415
331.19034
MS (MB)
m/z

(i)

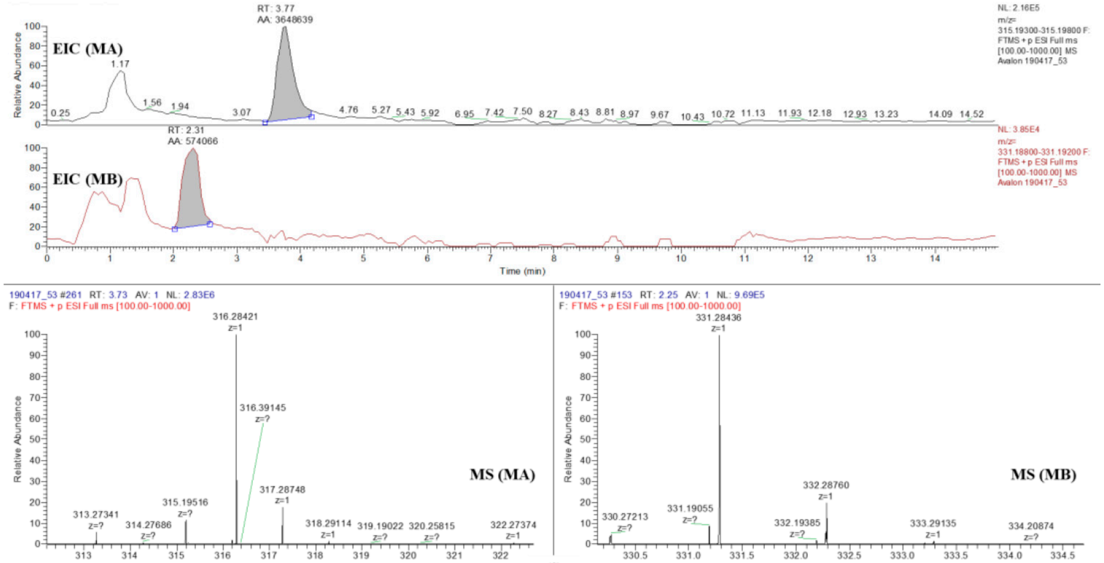
EIC (MA)
RT: 3.77
AA: 3648639
NL: 2.16E5
m/z= 315.19300-315.19800 F: FTMS + p ESI Full ms [100.00-1000.00] MS Avalon 190417_53
EIC (MB)
RT: 2.31
AA: 574066
NL: 3.85E4
m/z= 331.18800-331.19200 F: FTMS + p ESI Full ms [100.00-1000.00] MS Avalon 190417_53
Relative Abundance
Time (min)
190417_53 #261 RT: 3.73 AV: 1 NL: 2.83E6
F: FTMS + p ESI Full ms [100.00-1000.00]
316.28421
315.19516
MS (MA)
190417_53 #153 RT: 2.25 AV: 1 NL: 9.69E5
F: FTMS + p ESI Full ms [100.00-1000.00]
331.28436
331.19055
MS (MB)
m/z

(j)

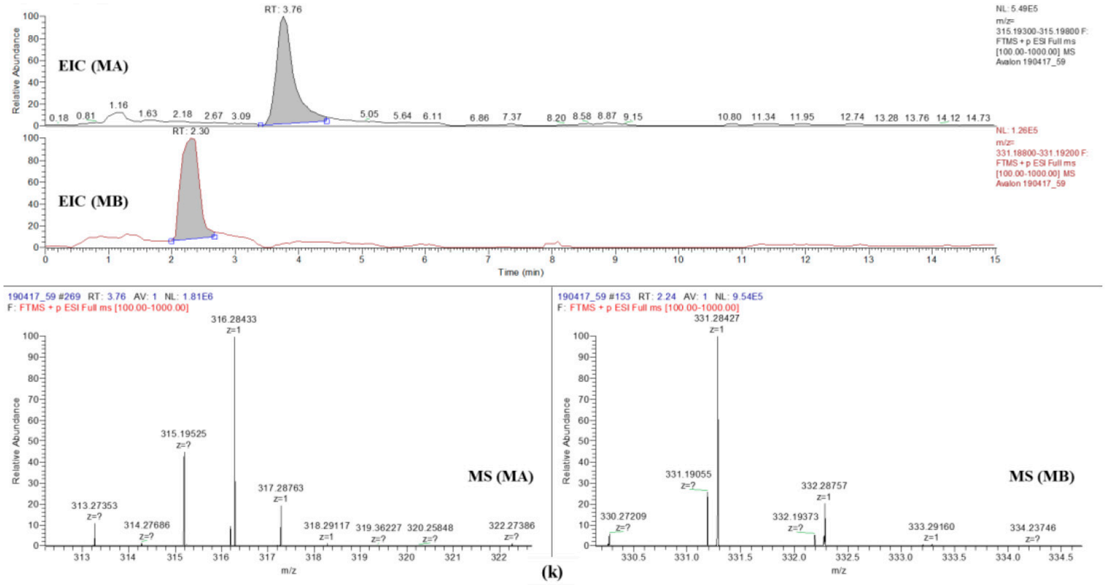


(k)

**Figure S2.** Extracted ion chromatograms (EIC) and mass spectra (MS) of MA and MB in rice grain extracts by UPLC-ESI-MS. (a), standard MAB; (b), Ko: Koshihikari; (c), KoCo: cooked Koshihikari; (d), KT1: shinnosuke rice; (e), KT2: seiten no hekireki rice; (f), KT3: ginga no shizuku rice; (g), KT4: ho no mai; (h), Bin 9; (i), KD18: Khang dan 18; (j), ST24; (k), RVT.
